# Cross-Sectional Study of Location-Based Built Environments, Physical Activity, Dietary Intake, and Body Mass Index in Adult Twins

**DOI:** 10.3390/ijerph20064885

**Published:** 2023-03-10

**Authors:** Glen E. Duncan, Feiyang Sun, Ally R. Avery, Philip M. Hurvitz, Anne Vernez Moudon, Siny Tsang, Bethany D. Williams

**Affiliations:** 1 Department of Nutrition and Exercise Physiology, Elson S. Floyd College of Medicine, Washington State University Health Sciences Spokane, Spokane, WA 99202, USA; 2 Department of Urban Design and Planning, College of Built Environments, University of Washington, Seattle, WA 98195, USA; 3 Center for Studies in Demography & Ecology, College of Arts and Sciences, University of Washington, Seattle, WA 98195, USA

**Keywords:** GPS, lifestyle behaviors, spatial measurement, walkability

## Abstract

We examined relationships between walkability and health behaviors between and within identical twin pairs, considering both home (neighborhood) walkability and each twin’s measured activity space. Continuous activity and location data (via accelerometry and GPS) were obtained in 79 pairs over 2 weeks. Walkability was estimated using Walk Score^®^ (WS); home WS refers to neighborhood walkability, and GPS WS refers to the mean of individual WSs matched to every GPS point collected by each participant. GPS WS was assessed within (WHN) and out of the neighborhood (OHN), using 1-mile Euclidean (air1mi) and network (net1mi) buffers. Outcomes included walking and moderate-to-vigorous physical activity (MVPA) bouts, dietary energy density (DED), and BMI. Home WS was associated with WHN GPS WS (*b* = 0.71, *SE* = 0.03, *p* < 0.001 for air1mi; *b* = 0.79, *SE* = 0.03, *p* < 0.001 for net1mi), and OHN GPS WS (*b* = 0.18, *SE* = 0.04, *p* < 0.001 for air1mi; *b* = 0.22, *SE* = 0.04, *p* < 0.001 for net1mi). Quasi-causal relationships (within-twin) were observed for home and GPS WS with walking (*ps* < 0.01), but not MVPA, DED, or BMI. Results support previous literature that neighborhood walkability has a positive influence on walking.

## 1. Introduction

Diet and physical activity behaviors are influenced by multiple interacting factors ranging from biology to policy [1]. Aspects of built environments (BE) influence health behaviors, and, subsequently, obesity and associated chronic diseases [2,3,4]. Specifically, a dearth of health-promoting BEs, characterized by a lack of access to food retail outlets providing healthy foods, poor walkability, and fewer outdoor park and recreational facilities accessible in proximity to home, is inversely associated with healthful dietary behaviors [5,6] and physical activity levels [7,8] across all ages. Interventions that target these upstream community-level factors are proposed as cost-effective means to promote and improve health across populations [9]. For these reasons, research informing prevention efforts should shift focus from individual-level factors to macro-level factors such as the BE to guide policies that influence all people in their daily lives.

The relationships between neighborhood BE and energy balance behaviors are nuanced and complex [10,11]. Self-selection bias, structural confounding, and measurement issues have hampered the neighborhood-effects literature [12]. Identical twins can be used as “quasi-experimental” controls of genetic and shared environmental factors (i.e., between-family confounds) that influence neighborhood selection, behaviors, and health [12,13,14,15]. As such, findings from twin analyses in genetically informed samples are considered “quasi-causal,” where the effect of the predictor on the outcome controls for any common genetic and familial environmental background they share and is, therefore, a partial approximation of any causal effect of the predictor [12,13,14,15]. Utilizing the twin study design to determine the relative influence of between-family factors, this study examined the between- and within-twin pair associations among the BE and health behaviors in identical twin pairs. For environmental exposures, the focus was on comparing the home neighborhood, which has been at the core of much of past research, and the total activity space experienced by individuals on a daily basis. We addressed the following research questions: (1) Do individuals who reside in more walkable neighborhoods spend more time in more walkable neighborhoods, including those not necessarily their own?; (2) Do individuals who reside in more walkable neighborhoods or spend more time in more walkable neighborhoods have lower dietary energy density and higher physical activity levels?; and (3) Do individuals who reside in more walkable neighborhoods or spend more time in more walkable neighborhoods have lower BMI?

## 2. Materials and Methods

### 2.1. Study Design and Participants

This was a cross-sectional study of objective measures of physical activity amounts and locations among individuals living in the Puget Sound region around Seattle, WA, USA. Participants wore a Qstarz BT-Q1000XT GPS data logger (Qstarz International Co. Ltd., Taipei, Taiwan) and Actigraph GT3X+ accelerometer (Actigraph Inc., Pensacola, FL, USA) attached to an elastic belt worn around the waist to collect data under free-living conditions over a 2-week period. Validated surveys were used to collect information on demographic and health characteristics, as well as dietary intake. Data were collected during 2012–2015. To retain only high-quality data, the GPS data were censored to keep only those points that were recorded with Standard Positioning Service (SPS) or Differential GPS (DGPS), and that had a horizontal dilution of precision (HDOP) < 6.

The initial sample included 187 identical twin participants from the community-based Washington State Twin Registry (WSTR). Recruitment procedures and details about the WSTR are reported elsewhere [16,17]. Recruited twins completed an in-person study visit followed by a 2-week remote data collection protocol. Inclusion criteria were living at the primary residence for at least 1 year and absence of physical conditions that limited mobility. The final sample consisted of 79 twin pairs (*n* = 158) who had complete data and met the minimum criteria for valid monitor “wear time”, defined as a minimum of 10 h per day of accelerometry wearing for 10 days [18] and any GPS measurement on each day. On average, participants wore both monitoring devices for 10.2 ± 3.1 days (median = 11, range = 2 to 14). Note that the lower end of the range indicates a participant who wore the accelerometer for the appropriate number of days but may have lacked GPS data on each of those days.

### 2.2. Exposures

The primary exposure was neighborhood walkability, a measure of the BE estimated using Walk Score^®^ (WS, Seattle, WA, USA) [19]. Each twin’s residential address and each GPS location coordinate were quantified using WS, which uses data from business listings, road networks, schools, and public transit to map walking distance to amenities in nine different categories weighted by importance [20]. Specific categories of points of interest include grocery stores, restaurants, coffee shops, banks, parks, schools, books (store or library), and various shopping and entertainment venues. The unpublished, proprietary algorithm then uses distances, counts, and weights to create a continuous score normalized on a scale of 0–100, with 0 representing the least and 100 being the most “walkable” neighborhoods [20]. This index is a valid measure of walkability [21]. In this study, WS values were used to classify home neighborhoods into five categories of neighborhood type: (1) Car dependent I (0–24), (2) Car dependent II (25–49), (3) Somewhat walkable (50–69), (4) Very walkable (70–89), and (5) Walker’s paradise (90–100).

“Home WS” refers to WS corresponding to a participant’s home address, and “GPS WS” refers to WS values corresponding to logged GPS coordinates for that participant’s wear time over the two-week assessment. The GPS WS values were calculated for all coordinate points logged for that participant’s wear time (i.e., including walking and non-walking) and for walking bouts only (i.e., bouts with a median speed between 2 km/h to 6 km/h, see description of physical activity outcomes below). Further descriptions of GPS WS calculations follow.

GPS WS was presented and analyzed as either a “within home” neighborhood (WHN) or an “out of home” neighborhood (OHN). The WHN GPS WS variables were assessed by averaging the walkability of GPS points that occurred within two defined neighborhood buffers surrounding the participant’s home address: (1) a 1-mile Euclidean (i.e., air1mi), and (2) a 1-mile network (i.e., net1mi) [22,23]. The OHN GPS WS variables were created by averaging the walkability of GPS points that occurred outside of those defined home neighborhood proximities.

GPS WS was also presented and analyzed as “unweighted” or “weighted” values. The unweighted mean is the average WS of all GPS points. The weighted mean was weighted for the duration of time spent in each location and is the sum of WS values associated with each GPS point multiplied by the duration of the corresponding GPS point, divided by the total duration. The duration was defined as the average of the lead and lag time for each GPS point.

### 2.3. Outcomes

Primary outcomes included diet quality (i.e., energy density), physical activity levels, and body mass index (BMI). The dietary energy density (DED) was assessed using a validated food-frequency questionnaire (FFQ), calculated by dividing the energy content of food items (in kilocalories) by the weight of food items (in grams) consumed. Two indicators of DED were analyzed: (1) only food, and (2) food and caloric beverages. The FFQ was developed by the Nutrition Assessment Shared Resource (NASR) of Fred Hutchinson Cancer Research Center (Seattle, WA, USA). Nutrient calculations were performed using the Nutrient Data System for Research (NDSR) software version 2017, developed by the Nutrition Coordinating Center, University of Minnesota, Minneapolis, MN, USA.

Physical activity levels were operationalized as walking bout minutes per week and moderate-to-vigorous physical activity (MVPA) bout minutes per week. Walking bouts were identified using a classification algorithm adapted from Kang et al. [24], described previously [25], and in brief below. MVPA bouts were identified as sustained intervals with 3D vector magnitude ≥ 2690 counts per minute (CPM) [26], using a modified 10-min bout definition that allows for up to two minutes outside the specified CPM threshold [18]. Accelerometry and GPS devices were configured to record data at 30-s intervals; data were combined into “LifeLogs” using common time stamps [11,27]. Light-to-moderate physical activity (LMPA) bouts used vector magnitude thresholds between 2000 and 6166 CPM. Walking bouts were identified as the subset of LMPA bouts that had (1) at least three records with GPS coordinates, (2) ≥20% of records with GPS coordinates, (3) median Doppler shift-based GPS speed between 2–6 kmh^−1^, and (4) spatial configuration. The spatial configuration criterion calculates the inter-point distance for all GPS coordinates in the bout and creates a minimum bounding circle (MBC) around the 95% most tightly clustered points in the bout; bouts with MBC > 20 m that met all other criteria were flagged as walking.

BMI was calculated from directly measured height and weight at a single in-person visit and expressed as kg/m^2^.

### 2.4. Covariates

Participants’ age, sex, education level, and annual household income were used as covariates in the statistical analyses. Age was computed based on the reported date of birth. Sex was self-reported as male or female. Education level refers to the highest level of education, ranging from less than high school to a graduate/professional degree. This variable was recorded to categorize individuals as having a bachelor’s degree or above or not. Annual household income was self-reported in eight categories, ranging from “less than $20,000” to “$80,000 or more.” Household income was recoded into two categories for analysis: “less than $50,000,” and “$50,000 or more.” 

### 2.5. Statistical Analysis

All statistical analyses were performed in the statistical program R 4.0.2. Descriptive statistics are presented as means and standard deviations (for continuous variables) and counts and proportions (for categorical variables). 

*Addressing research question 1*, a series of linear mixed-effects models (LMMs) were used to examine the associations between home WS and GPS WS. For the LMMs, we first examined the association between an individual’s home WS and the average GPS WS. A random intercept was included to account for correlations between members of a twin pair, but not controlling for pair-level confounds. This is referred to as “phenotypic associations” (Model 1). Next, we included the mean home WS between twin pairs into the LMMs to estimate the average GPS WS (Model 2). A random intercept was included to control for within-pair correlations. These models control for the between-family confounds of the relationship between the home WS and the average GPS WS. The regression coefficient for the individual-level predictor represents the “quasi-causal” effect of the predictor on the outcome, controlling for between-pair confounds. The final sets of models further investigated the potential between-pair confounds, by including participants’ age, sex, education level (bachelor’s degree or above or not), and annual household income ($50,000 or more) into the LMMs (Model 3). 

*Addressing research questions 2 and 3*, similar sets of LMMs were performed to investigate the association between WS and DED, WS and physical activity outcomes (walking and MVPA), and WS and BMI. Physical activity was square root transformed, and BMI was log-transformed. Home and GPS WS were divided by 100 to allow variables to be on similar scales. Separate analyses were conducted for WHN versus OHN GPS WS, and unweighted versus duration-weighted GPS WS.

For each analysis, the Bonferroni correction method was applied to control for Type I error rates. Adjusted *p*-values are indicated in the footnotes of each table.

## 3. Results

Descriptive statistics of selected demographic characteristics and primary variables of interest are shown in Table 1. The age of the sample ranged from 23.4 to 75.3 years old (*M* = 44.6, *SD* = 12.4). Most participants self-identified as White (85%) and non-Hispanic (95.1%). In general, participants spent the highest proportion of their WHN time (27%) and OHN time (26%) in neighborhoods considered Very walkable (Supplementary Table S1). Supplementary Figure S1 illustrates the general study area and provides an example of a very walkable (left side) and car-dependent (right side) neighborhood.

*Addressing research question 1*, Table 2 provides results from the LMMs estimating the associations between home WS and GPS WS. Results for Model 1 showed positive associations between home WS and WHN GPS WS (*b* = 0.71, *SE* = 0.03, *p* < 0.001 for air1mi; *b* = 0.79, *SE* = 0.03, *p* < 0.001 for net1mi), as well as OHN GPS WS (*b* = 0.18, *SE* = 0.04, *p* < 0.001 for air1mi; *b* = 0.22, *SE* = 0.04, *p* < 0.001 for net1mi). Thus, participants who reside in more walkable neighborhoods are more likely to spend more time in more walkable neighborhoods, both within and outside of their own home neighborhoods. Model 2 showed a quasi-causal effect of home WS on the average WHN GPS WS (*b* = 0.63, *SE* = 0.05, *p* < 0.001 for air1mi; *b* = 0.76, *SE* = 0.05, *p* < 0.001 for net1mi); thus, living in a more walkable neighborhood is associated with increased time spent in nearby walkable neighborhoods, after controlling for between-pair confounds. In contrast, results were attenuated and became non-significant in Model 2 for OHN GPS WS (*b* = 0.08, *SE* = 0.05, *p* = 0.140 for air1mi; *b* = 0.10, *SE* = 0.05, *p* = 0.063 for net1mi); thus, the association between home walkability and time spent in walkable areas outside of participants’ home neighborhoods is mediated by between-pair confounds. Results remained similar after accounting for additional confounding variables (Model 3). Comparable results were observed for weighted GPS WS. 

*Addressing research question 2*, Table 3, Table 4 and Table 5 present results of LMMs determining associations between WS with DED and physical activity. As shown in Table 3, there was no significant association between home WS and the two primary DED outcomes (Model 1). Results remained consistent when accounting for between-pair confounds (Model 2) and additional confounders (Model 3). Comparable results were observed when examining the associations between GPS WS and DED, across all buffer types, WHN versus OHN GPS WS, and unweighted versus duration-weighted GPS WS, across all Models (Table 4).

Table 3 also shows results estimating the association between home WS and physical activity. There is a positive association between home WS with walking (*b* = 4.89, *SE* = −1.21, *p* < 0.001) and MVPA (*b* = 3.03, *SE* = 1.44, *p* = 0.037) (Model 1). Thus, individuals who reside in more walkable neighborhoods are more likely to have higher levels of physical activity. When between-pair confounds are included in Model 2, only the association between home WS and walking remained statistically significant (*b* = 5.56, *SE* = 1.72, *p* < 0.001), providing evidence for a quasi-causal effect of home WS on the amount of walking. However, there is no quasi-causal effect of home WS on the amount of MVPA (*b* = 2.83, *SE* = 1.96, *p* = 0.150). Results remained similar for both walking and MVPA after adjusting for additional confounders (Model 3). 

Comparable results were observed when examining the associations between unweighted GPS WS and physical activity (Table 5). Few significant associations were observed between duration-weighted WHN GPS WS and physical activity outcomes across all models. Quasi-causal associations were observed between duration-weighted OHN GPS WS with walking (*b* = 11.09, *SE* = 3.47, *p* = 0.002 for air1mi; *b* = 10.17, *SE* = 3.72, *p* = 0.008 for net1mi), but not MVPA when adjusting significance level for multiple comparisons (*b* = 10.62, *SE* = 3.86, *p* = 0.007 for air1mi; *b* = 10.83, *SE* = 4.14, *p* = 0.011 for net1mi) (Model 2). Results remained similar after accounting for additional confounding variables (Model 3). 

*Addressing research question 3*, Table 3 and Table 6 present results of LMMs determining associations between WS with BMI. In summary, there was no significant phenotypic association between home or GPS WS with BMI.

## 4. Discussion

In this research, we investigated the effects of the BE on lifestyle behaviors in a community-based sample of adult identical twins who were reared together but now live apart. This unique sample allowed us to examine environmental influences on health-related outcomes, controlling for the between-family effects that might otherwise introduce selection biases into the choice of living environments. Specifically, the present study results provide novel insight into comparing the relationships between walkability measured at the location of individuals’ homes and at their actual daily locations with health behaviors, including physical activity and diet. Results suggest that individuals who reside in more walkable neighborhoods are more likely to spend more time in more walkable neighborhoods overall. Findings related to home walkability and spending time in walkable neighborhoods close to home remained significant in fully adjusted models (i.e., Model 3) and were thus quasi-causal, whereas the effect of time (i.e., weighted GPS) spent in walkable areas outside of the home neighborhood was confounded by between-family factors. Quasi-causal associations between both home and GPS WS with the amount of weekly walking were observed in fully adjusted models, but not with MVPA. Walkability surrounding participants’ homes and GPS locations were not associated with DED, a proxy measure of diet quality, or BMI. The current project offers innovative solutions to control confounding influences surrounding the association of BE with health. It builds on previous work by considering the BE over each twin’s objectively measured activity space—the environment in which they live, work, and spend time daily.

Present findings indicate that those who reside in more walkable neighborhoods were more likely to spend time in more walkable neighborhoods, especially in locations near home. This novel finding objectively verifies a common assumption among many geographic and BE studies that the home BE affects behavior. Results also indicate that those who live and spend time in nearby walkable neighborhoods exhibit higher levels of walking. These findings support the larger body of literature identifying BE factors to be predictive of community physical activity. However, some studies have reported conflicting evidence, particularly in determining causal mechanisms [2,3,4]. For example, prior cross-sectional studies have shown that densities of community resources for a healthful diet and physical activity (e.g., food sources and greenspaces) were positively related to diet quality/composition [28,29] and physical activity measures [30,31]. In contrast, a review of neighborhood-based natural experiments reported a lack of longitudinal evidence that change in neighborhood BE was related to a change in residents’ diets and physical activity behaviors [32]. It should be noted that even minor changes to health behaviors attributable to the BE at the individual level have the potential to improve health outcomes at the population level [32,33].

In the current sample, neither participants’ homes nor GPS WS were related to levels of MVPA after adjusting for between family and demographic confounders. This aligns with previous research focused on BE influences on the location-based physical activity performed at higher intensities [34,35], including previous studies with the WSTR [13]. It expands on this work by introducing a location- and duration-based means to assess regular exposure to BE. Multiple study results indicated that associations between walkability features and MVPA were only significant when predictors and outcomes were located within the same geographic buffer [34,35]. Specifically, BE constructs surrounding the home were related to MVPA performed in the home neighborhood, but not total MVPA [34,35]. Importantly, MVPA was also reported to be performed more frequently outside of home and work neighborhoods compared to within [34]. Some studies suggest additional community-related factors drive the relationship between neighborhood environment and MVPA, such as perceived neighborhood safety [36] and greenspace [37]. This said, conflicting findings exist linking BE variables with location-based MVPA [36,37,38]. As is common in geospatial research, discrepancies in study findings may be attributed to nuanced differences in BE assessment, differing social constructs of geographic locations across studies, and differences in measures of health outcomes. Overall, the present study findings, in combination with previous literature, suggest the continued need for careful consideration of the spatial context in relation to health behaviors. Further qualitative and mixed-methods research may be needed to fully understand how individuals interact within their living and working environment, and location-based drivers of MVPA.

In the current sample, the participant’s home and GPS WS were not related to DED or BMI. Previous studies have suggested that, especially in areas with a higher density of unhealthy food outlets, increased walkability may increase access to less healthful, energy-dense fast foods, and thus may have a negative impact on dietary outcomes [39,40] that could potentially nullify health benefits of either increased supermarket access or physical activity opportunities. There is substantial evidence to confirm that individuals residing in and traveling through neighborhoods with a high density of unhealthy food outlets consume more energy-dense foods and fewer fresh fruits and vegetables [5,41,42]. The current study utilized Walk Score^®^ as a measure of the BE, which scored participants’ residences and actual locations primarily based on the surrounding density of businesses, but without necessarily considering the differential influence of specific business types (i.e., grocery versus parks versus entertainment). Given the previously confirmed associations between food access and physical activity behaviors with dietary intake, future studies could expand on this work by examining how standard BE features (i.e., street connectivity, population density, public transit, etc.) are spatially related to health-promoting food resources, and further investigate their independent and cumulative relationships with a variety of outcomes, including BMI.

There are several strengths of the current study. First, the combined use of an accelerometer and GPS to determine physical activity allowed for precise and objective measurement of physical activity behaviors, including type, amount, and geospatial location. The assessment period being two weeks in duration is a study strength [34,43], and further included both weekdays and weekends, which is necessary to provide an accurate reflection of a person’s “usual” activity [44,45]. GPS WS were analyzed using two different buffer types (Euclidean and network), Within versus Out of home WS, and as both unweighted and duration weighted to allow for comparison and more precise interpretation of BE influences. Our scientific approach is genetically informed and integrates conceptual models from the behavioral and social sciences with biological, computational, and physical measures, and thus considers both between-family confounds and standard demographic control variables. 

Discussion of study weaknesses is warranted. It should be noted that although the twin design can effectively control for between-family confounds and is considered as “quasi-causal,” causality—especially reverse causality—cannot necessarily be inferred due to the cross-sectional design. The sample was primarily female (72.2%), White (85.0%), and educated at the bachelor’s level and above (78.9%); thus, the generalizability of the study findings is limited. Dietary outcomes were self-reported, and as such, are subject to social desirability and other biases. The commercially available Walk Score^®^ algorithm for estimating walkability is primarily an index of utilitarian destinations accessible by walking and does not equally include other measures of urban form that could influence physical activity; furthermore, the proprietary nature of the Walk Score^®^ source data and algorithm reduce interpretability. Finally, while accelerometers record continuously, cold starts and signal impedance from urban canyons or other obstructions may result in incomplete data from GPS data loggers, leading to underestimation of the duration spent in various locations.

## 5. Conclusions

In conclusion, the present study coupled innovative technology with advanced methods in geospatial data analysis and integrated spatial databases to determine relationships between the BE with health behaviors in a real-time and space continuum. Specifically, we investigated the effects of the BE on DED, walking, MVPA, and BMI in a community-based sample of adult identical twins to examine environmental influences on health-related outcomes independent of between-family confounders. A quasi-causal relationship was found between home neighborhood walkability with time spent in nearby walkable neighborhoods. Individuals who resided in and spent time in more walkable neighborhoods were more likely to exhibit higher levels of physical activity; relationships remained significant for walking, but not for MVPA, after adjusting for between-family confounds and demographic covariates. Walkability surrounding participants’ homes and GPS locations were not associated with DED or BMI. Urban policy and city planning should continue to promote neighborhood BE to improve walking and related community-level health outcomes. Future studies can build on this work by further investigating the importance of combined diet- and walk-related BE, and community-related drivers of physical activity modes performed at a higher intensity.

## Figures and Tables

**Table 1:** Descriptive statistics for demographic characteristics and primary variables of interest among identical twin pairs (*n* = 158).

	M	SD	Med	Min	Max
Age	44.6	12.4	39.7	23.4	75.3
BMI (kg/m^2^)	26.6	5.9	25.3	17.2	53.9
	n	%			
Sex					
Male	44	27.8			
Female	114	72.2			
Race/ethnicity					
White	134	85.0			
Non-Hispanic	150	95.1			
Marital status					
Single	32	20.2			
Married/Living with partner	107	67.6			
Separated/Divorced	15	9.5			
Widowed	4	2.5			
Highest level of education					
Less than high school	1	0.6			
High school/GED level	7	4.4			
Some college/Associates degree	25	15.9			
Bachelor’s degree or above	124	78.9			
Household income					
$50,000 or more	129	0.8			
	**M**	**SD**	**Med**	**Min**	**Max**
Home WS	57.4	26.7	61.0	3.0	98.0
GPS WS					
Within home					
air1mi	60.7	21.9	59.4	15.2	96.7
net1mi	61.1	23.7	62.3	14.4	96.9
Out of home					
air1mi	63.2	14.5	63.3	24.3	90.9
net1mi	63.2	14.8	63.3	25.8	91.6
GPS WS (weighted)					
Within home					
air1mi	61.1	23.0	60.5	15.2	138.8
net1mi	62.1	28.9	62.0	14.3	259.0
Out of home					
air1mi	63.3	14.9	63.4	24.2	97.9
net1mi	63.4	15.0	63.2	25.7	91.3
GPS WS—walking bouts only					
Within home					
air1mi	64.2	24.6	71.0	15.1	98.0
net1mi	65.6	24.0	71.5	15.8	98.0
Out of home					
air1mi	70.8	23.9	79.0	13.6	99
net1mi	68.8	23.6	73.9	13.6	99
GPS WS (weighted)—walking bouts only					
Within home					
air1mi	63.9	24.8	70.2	15.1	106.0
net1mi	69.5	41.2	72.4	15.5	377.0
Out of home					
air1mi	71.6	25.0	78.4	13.1	130.5
net1mi	69.1	23.9	73.9	10.3	103.4
Dietary energy density (kcal/grams) *					
Food only	1.4	0.3	1.4	0.8	2.4
Food & caloric beverages	1.2	0.3	1.1	0.6	1.9
Physical activity					
Walking (min/wk)	59.1	68.0	36.0	0.0	418.6
MVPA (min/wk)	131.1	124.2	102.2	0.0	656.8

WS = WalkScore. air1mi = 1-mile air (Euclidean) buffer type. net1mi = 1-mile network buffer type. MVPA = Moderate to Vigorous Physical Activity. * Dietary energy density was assessed using a validated food-frequency questionnaire (FFQ), calculated by dividing the energy content of food items (in kilocalories) by the weight of food items (in grams) consumed.

**Table 2:** Unstandardized estimates from linear mixed-effects models estimating the associations between home WalkScore and GPS WalkScore (unweighted and weighted).

	Individual Home WS			
	Estimate	SE	95%CI	
GPS WS				
Model 1				
Within home				
air1mi	0.71 *	0.03	0.65	0.77
net1mi	0.79 *	0.03	0.73	0.85
Out of home				
air1mi	0.18 *	0.04	0.10	0.25
net1mi	0.22 *	0.04	0.14	0.29
Model 2				
Within home				
air1mi	0.63 *	0.05	0.53	0.72
net1mi	0.76 *	0.05	0.66	0.85
Out of home				
air1mi	0.08	0.05	−0.03	0.18
net1mi	0.10	0.05	0	0.20
Model 3				
Within home				
air1mi	0.64 *	0.05	0.55	0.73
net1mi	0.76 *	0.05	0.67	0.85
Out of home				
air1mi	0.08	0.05	−0.03	0.18
net1mi	0.10	0.05	0	0.20
GPS WS (weighted)				
Model 1				
Within home				
air1mi	0.73 *	0.03	0.67	0.79
net1mi	0.79 *	0.05	0.68	0.89
Out of home				
air1mi	0.19 *	0.04	0.11	0.27
net1mi	0.22 *	0.04	0.15	0.30
Model 2				
Within home				
air1mi	0.66 *	0.05	0.56	0.77
net1mi	0.82 *	0.09	0.65	0.99
Out of home				
air1mi	0.10	0.05	0	0.21
net1mi	0.12	0.05	0.01	0.22
Model 3				
Within home				
air1mi	0.68 *	0.05	0.58	0.78
net1mi	0.83 *	0.09	0.65	1.00
Out of home				
air1mi	0.10	0.06	-0.01	0.21
net1mi	0.12	0.05	0.01	0.23

* Indicates statistically significant association. WS, WalkScore. air1mi = 1-mile air (Euclidean) buffer type. net1mi = 1-mile network buffer type. Home and GPS WS were divided by 100 to allow variables to be on similar scales. Bonferroni correction method applied; significance level set at *p* < 0.006. Model 1: Associations between the individual’s home WS and the average GPS WS with a random intercept included to take into account correlations between members of a pair, but not controlling for pair-level confounds (i.e., “phenotypic associations”). Model 2: LMMs from Model 1, also including the mean home WS between twin pairs and a random intercept to control for within-pair correlations, thus controlling for the between-pair (i.e., genetics and shared environmental) confounds. The regression coefficient for the individual-level predictor represents the “quasi-causal” effect of the predictor on the outcome, controlling for between-pair confounds. Model 3: LMMs from Model 2, also including participants’ age, sex, education level (Bachelor’s degree or not), and annual household income ($50,000 or more).

**Table 3:** Unstandardized estimates from linear mixed-effects models estimating the associations between home WalkScore with dietary energy density, physical activity, and body mass index.

	Individual Home WS			
	Estimate	SE	95%CI	
Dietary Energy Density				
Model 1				
Food only	−0.01	0.08	−0.16	0.14
Food & caloric beverages	−0.04	0.07	−0.17	0.09
Model 2				
Food only	0.07	0.10	−0.13	0.26
Food & caloric beverages	−0.01	0.09	−0.18	0.17
Model 3				
Food only	0.08	0.10	−0.12	0.28
Food & caloric beverages	0.01	0.08	−0.16	0.17
Physical Activity				
Model 1				
Walking	4.89	1.21	2.52	7.26
MVPA	3.03	1.44	0.20	5.87
Model 2				
Walking	5.56	1.72	2.17	8.95
MVPA	2.83	1.96	−1.04	6.69
Model 3				
Walking	5.86	1.66	2.62	9.12
MVPA	3.12	2.01	−0.81	7.05
Body Mass Index				
Model 1	−0.04	0.03	−0.11	0.02
Model 2	−0.01	0.04	−0.08	0.07
Model 3	−0.01	0.05	−0.07	0.07

WS = WalkScore. Home WS was divided by 100. MVPA = Moderate to Vigorous Physical Activity. Physical activity was square root transformed. BMI = Body Mass Index. BMI was log-transformed. Bonferroni correction method applied; significance level set at *p* < 0.007. Model 1: Associations between an individual’s home walkscore and the outcome with a random intercept included to consider correlations between members of a pair, but not controlling for pair-level confounds (i.e., “phenotypic associations”). Model 2: LMMs from Model 1, also including the mean home WS between twin pairs and a random intercept to control for within-pair correlations, thus controlling for the between-pair (i.e., genetics and shared environmental) confounds. The regression coefficient for the individual-level predictor represents the “quasi-causal” effect of the predictor on the outcome, controlling for between-pair confounds. Model 3: LMMs from Model 2, also including participants’ age, sex, education level (Bachelor’s degree or not), and annual household income ($50,000 or more).

**Table 4:** Unstandardized estimates from linear mixed-effects models estimating the associations between dietary energy density and GPS WalkScore (unweighted and weighted).

	GPS WS (Unweighted)															
	Within Home								Out of Home							
	air1mi				net1mi				air1mi				net1mi			
	Estimate	SE	95%CI		Estimate	SE	95%CI		Estimate	SE	95%CI		Estimate	SE	95%CI	
Model 1																
Food only	−0.08	0.09	−0.26	0.11	−0.06	0.09	−0.23	0.12	−0.21	0.15	−0.50	0.08	−0.21	0.14	−0.49	0.08
Food & caloric beverages	−0.08	0.08	−0.23	0.07	−0.06	0.07	−0.20	0.08	−0.1	0.12	−0.35	0.14	−0.05	0.12	−0.28	0.18
Model 2																
Food only	0.10	0.13	−0.16	0.35	0.09	0.12	−0.16	0.33	−0.02	0.21	−0.44	0.40	0.02	0.23	−0.43	0.48
Food & caloric beverages	−0.05	0.11	−0.26	0.17	−0.01	0.1	−0.20	0.17	−0.02	0.19	−0.39	0.35	0.05	0.18	−0.30	0.41
Model 3																
Food only	0.13	0.13	−0.12	0.38	0.12	0.12	−0.12	0.36	0.00	0.22	−0.43	0.43	0.05	0.23	−0.41	0.51
Food & caloric beverages	−0.04	0.11	−0.25	0.17	−0.02	0.09	−0.21	0.16	0.00	0.18	−0.36	0.35	0.02	0.18	−0.33	0.36
	**GPS WS (weighted)**															
	**Within home**								**Out of home**							
	**air1mi**				**net1mi**				**air1mi**				**net1mi**			
	**Estimate**	**SE**	**95%CI**		**Estimate**	**SE**	**95%CI**		**Estimate**	**SE**	**95%CI**		**Estimate**	**SE**	**95%CI**	
Model 1																
Food only	−0.05	0.09	−0.23	0.12	−0.11	0.07	−0.26	0.03	−0.17	0.14	−0.45	0.11	−0.14	0.14	−0.43	0.14
Food & caloric beverages	−0.07	0.07	−0.22	0.07	−0.09	0.06	−0.20	0.02	−0.07	0.12	−0.31	0.16	−0.02	0.11	−0.25	0.20
Model 2																
Food only	0.11	0.12	−0.12	0.35	−0.02	0.09	−0.20	0.17	0.08	0.2	−0.32	0.47	0.20	0.22	−0.24	0.64
Food & caloric beverages	−0.04	0.10	−0.24	0.15	−0.04	0.07	−0.18	0.10	0.03	0.18	−0.32	0.39	0.11	0.18	−0.23	0.46
Model 3																
Food only	0.15	0.12	−0.09	0.38	0.00	0.09	−0.18	0.19	0.10	0.21	−0.31	0.52	0.22	0.22	−0.22	0.66
Food & caloric beverages	−0.04	0.10	−0.23	0.15	−0.05	0.07	−0.18	0.09	0.04	0.17	−0.30	0.38	0.08	0.17	−0.25	0.42

WS = WalkScore. air1mi = 1-mile air (Euclidean) buffer type. net1mi = 1-mile network buffer type. GPS WS was divided by 100. Bonferroni correction method applied; significance level set at *p* < 0.001. Model 1: Associations between the average GPS walkscore and dietary energy density with a random intercept included to take into account correlations between members of a pair, but not controlling for pair-level confounds (i.e., “phenotypic associations”). Model 2: LMMs from Model 1, also including the mean GPS walkscore between twin pairs and a random intercept to control for within-pair correlations, thus controlling for the between-pair (i.e., genetics and shared environmental) confounds. The regression coefficient for the individual-level predictor represents the “quasi-causal” effect of the predictor on the outcome, controlling for between-pair confounds. Model 3: LMMs from Model 2, also including participants’ age, sex, education level (Bachelor’s degree or not), and annual household income ($50,000 or more).

**Table 5:** Unstandardized estimates from linear mixed-effects models estimating the associations between physical activity and GPS WalkScore (unweighted and weighted).

	GPS WS (Unweighted)															
	Within Home								Out of Home							
	air1mi				net1mi				air1mi				net1mi			
	Estimate	SE	95%CI		Estimate	SE	95%CI		Estimate	SE	95%CI		Estimate	SE	95%CI	
Model 1																
Walking	5.24 *	1.50	2.31	8.18	4.55 *	1.42	1.77	7.33	8.49 *	2.29	3.98	12.98	8.43 *	2.33	3.86	12.98
MVPA	4.34	1.78	0.84	7.85	3.38	1.70	0.06	6.72	9.69 *	2.68	4.43	14.95	10.55 *	2.73	5.21	15.90
Model 2																
Walking	6.14	2.22	1.75	10.52	5.32	2.04	1.29	9.34	11.61 *	3.63	4.45	18.78	10.54	3.83	2.98	18.11
MVPA	4.33	2.54	−0.67	9.33	3.29	2.33	−1.30	7.88	10.59	4.06	2.59	18.59	11.75	4.25	3.37	20.12
Model 3																
Walking	6.17	2.17	1.92	10.42	5.15	2.01	1.19	9.08	11.34 *	3.59	4.31	18.36	11.05 *	3.75	3.69	18.39
MVPA	4.25	2.57	−0.79	9.28	3.02	2.35	−1.59	7.62	10.66	4.14	2.51	18.73	11.79	4.28	3.41	20.15
	**GPS WS (weighted)**															
	**Within home**								**Out of home**							
	**air1mi**				**net1mi**				**air1mi**				**net1mi**			
	**Estimate**	**SE**	**95%CI**		**Estimate**	**SE**	**95%CI**		**Estimate**	**SE**	**95%CI**		**Estimate**	**SE**	**95%CI**	
Model 1																
Walking	4.90 *	1.43	2.11	7.70	2.67	1.19	0.34	4.99	8.04 *	2.23	3.65	12.42	7.70 *	2.3	3.17	12.22
MVPA	3.93	1.69	0.62	7.26	1.32	1.40	−1.43	4.08	9.25 *	2.61	4.13	14.36	9.61 *	2.7	4.31	14.90
Model 2																
Walking	5.52	2.05	1.48	9.57	3.21	1.57	0.11	6.31	11.09 *	3.47	4.26	17.93	10.17	3.72	2.82	17.51
MVPA	4.30	2.33	−0.30	8.89	1.24	1.79	−2.29	4.77	10.62	3.86	3.02	18.22	10.83	4.14	2.66	19.00
Model 3																
Walking	5.54	2.00	1.61	9.47	3.05	1.57	−0.03	6.12	10.98 *	3.41	4.28	17.66	10.52	3.65	3.35	17.66
MVPA	4.18	2.37	−0.46	8.81	1.00	1.81	−2.55	4.55	10.63	3.93	2.88	18.30	10.88	4.17	2.72	19.04

* Indicates statistically significant association. WS = WalkScore. air1mi = 1-mile air (Euclidean) buffer type. net1mi = 1-mile network buffer type. MVPA = Moderate to Vigorous Physical Activity. Physical activity was square root transformed. GPS WS was divided by 100. Bonferroni correction method applied; significance level set at *p* < 0.001. Model 1: Associations between the average GPS walkscore and physical activity with a random intercept included to take into account correlations between members of a pair, but not controlling for pair-level confounds (i.e., “phenotypic associations”). Model 2: LMMs from Model 1, also including the mean GPS walkscore between twin pairs and a random intercept to control for within-pair correlations, thus controlling for the between-pair (i.e., genetics and shared environmental) confounds. The regression coefficient for the individual-level predictor represents the “quasi-causal” effect of the predictor on the outcome, controlling for between-pair confounds. Model 3: LMMs from Model 2, also including participants’ age, sex, education level (Bachelor’s degree or not), and annual household income ($50,000 or more).

**Table 6:** Unstandardized estimates from linear mixed-effects models estimating the associations between body mass index and GPS WalkScore (unweighted and weighted).

	GPS WS (Unweighted)															
	Within Home								Out of Home							
	air1mi				net1mi				air1mi				net1mi			
	Estimate	SE	95%CI		Estimate	SE	95%CI		Estimate	SE	95%CI		Estimate	SE	95%CI	
Body Mass Index																
Model 1	−0.06	0.04	−0.14	0.02	−0.05	0.04	−0.13	0.03	0.03	0.07	−0.11	0.17	0.00	0.07	−0.14	0.14
Model 2	−0.01	0.05	−0.10	0.08	−0.02	0.04	−0.10	0.07	0.14	0.08	−0.01	0.29	0.12	0.08	−0.03	0.27
Model 3	−0.02	0.05	−0.11	0.09	−0.01	0.05	−0.10	0.08	0.12	0.07	−0.03	0.27	0.12	0.08	−0.04	0.27
	**GPS WS (weighted)**															
	**Within home**								**Out of home**							
	**air1mi**				**net1mi**				**air1mi**				**net1mi**			
	**Estimate**	**SE**	**95%CI**		**Estimate**	**SE**	**95%CI**		**Estimate**	**SE**	**95%CI**		**Estimate**	**SE**	**95%CI**	
Body Mass Index																
Model 1	−0.04	0.04	−0.12	0.03	−0.03	0.03	−0.09	0.03	0.03	0.07	−0.11	0.16	−0.01	0.07	−0.15	0.13
Model 2	−0.00	0.04	−0.09	0.08	−0.01	0.03	−0.08	0.05	0.12	0.07	−0.02	0.27	0.10	0.07	−0.05	0.24
Model 3	−0.01	0.04	−0.09	0.08	−0.02	0.03	−0.08	0.05	0.10	0.07	−0.03	0.24	0.09	0.08	−0.05	0.24

WS = WalkScore. BMI = Body Mass Index. BMI was log-transformed. air1mi = 1-mile air (Euclidean) buffer type. net1mi = 1-mile network buffer type. GPS WS was divided by 100. Bonferroni correction method applied; significance level set at *p* < 0.002. Model 1: Associations between the average GPS walkscore and body mass index with a random intercept included to take into account correlations between members of a pair, but not controlling for pair-level confounds (i.e., “phenotypic associations”). Model 2: LMMs from Model 1, also including the mean GPS walkscore between twin pairs and a random intercept to control for within-pair correlations, thus controlling for the between-pair (i.e., genetics and shared environmental) confounds. The regression coefficient for the individual-level predictor represents the “quasi-causal” effect of the predictor on the outcome, controlling for between-pair confounds. Model 3: LMMs from Model 2, also including participants’ age, sex, education level (Bachelor’s degree or not), and annual household income ($50,000 or more).

## Data Availability

The data supporting the results of the present study are owned by the Washington State Twin Registry (WSTR). Thus, the data cannot be publicly shared as it involves third-party data. However, researchers interested in applying to gain access to the data can do so by contacting the WSTR and completing the appropriate forms stipulated in the WSTR Policies & Procedures guidelines. Application information can be sent to the Scientific Operations Manager at the following URL (https://wstwinregistry.org/contact-us/, 1 March 2023) or via email (ws.twinregistry@wsu.edu). The authors confirm they did not have any special access privileges that others would not have.

## Supplementary Materials

The following supporting information can be downloaded at: https://www.mdpi.com/article/10.3390/ijerph20064885/s1, Figure S1: General study area in Seattle, WA, with inset maps of two illustrative neighborhoods; Table S1: Descriptive statistics for proportion of time spent in different neighborhoods, by neighborhood type, among identical twin pairs (*n* = 158).

## Abbreviations

Air1mi	1-mile Euclidean buffer
BE	Built Environment
BMI	Body mass index
CPM	Counts per minute
DED	Dietary energy density
FFQ	Food frequency questionnaire
LMM	Linear mixed-effects models
LMPA	Light to moderate physical activity
MBC	Minimum bounding circle
MVPA	Moderate to vigorous physical activity
NASR	Nutrition assessment shared resource
Net1mi	1-mile network buffer
OHN	Outside of home neighborhood
PA	Physical activity
WHN	Within home neighborhood
WS	WalkScore
WSTR	Washington state twin registry
