# Supplementary material for: Cross-Sectional Study of Location-Based Built Environments, Physical Activity, Dietary Intake, and Body Mass Index in Adult Twins
Source: Int J Environ Res Public Health. 2023 Mar 10;20(6):4885. doi: 10.3390/ijerph20064885 (PMC10049069; doi:10.3390/ijerph20064885)

Supplementary Table S1. Descriptive statistics for proportion of time spent in different neighborhoods, by neighborhood type, among identical twin pairs (*n*=158).

	GPS Proportion of Time Spent															
	Within home								Out of home							
Neighborhood Type	air1mi				net1mi				air1mi				net1mi			
	M	SD	Min	Max	M	SD	Min	Max	M	SD	Min	Max	M	SD	Min	Max
Car dependent I (0-24)	12.2	21.4	0.0	90.3	12.3	24.2	0.0	95.5	11.8	13.3	0.0	78.5	12.0	13.1	0.0	75.7
Car dependent II (25-49)	21.6	24.4	0.0	96.7	22.2	27.4	0.0	96.1	19.1	14.0	0.4	88.1	19.5	14.4	0.7	87.4
Somewhat walkable (50-69)	24.0	23.9	0.0	98.6	21.0	25.0	0.0	99.3	21.2	13.7	1.0	84.9	20.5	12.1	1.1	72.3
Very walkable (70-89)	26.7	28.1	0.0	86.7	27.5	30.3	0.0	90.6	25.5	15.6	1.0	77.9	25.5	15.7	1.4	78.8
Walker's paradise (90-100)	15.5	26.7	0.0	98.9	17.0	28.6	0.0	100.0	22.4	20.6	0.0	85.3	22.5	20.7	0.0	83.4

air1m = 1-mile air (Euclidean) buffer type. net1m = 1-mile network buffer type.

Supplementary Figure S1. General study area in Seattle, WA, with inset maps of two illustrative neighborhoods.

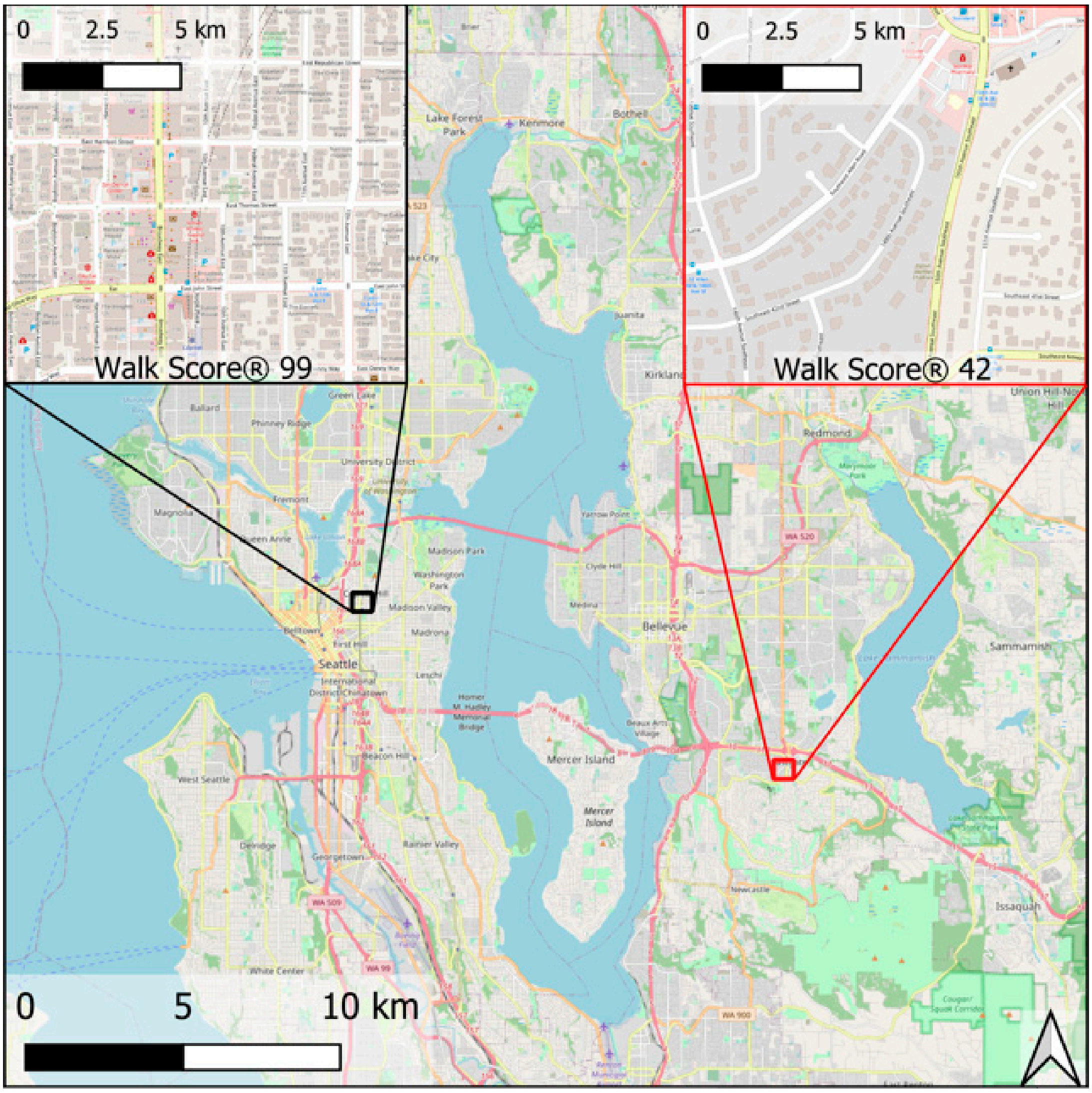

Supplement: Supplementary file 1 [file ijerph-20-04885-s001.zip › ijerph-2228462-supplementary.pdf]
